# Supplementary material for: Mutant SOD1 expressed by oligodendrocytes aggregates in myelinic nanochannels and accelerates disease progression in familial ALS mice
Source: bioRxiv. 2026 Jun 11:2026.06.09.731100. Preprint. [Version 1] doi: 10.64898/2026.06.09.731100 (PMC13277883; doi:10.64898/2026.06.09.731100)
Supplement: Supplementary file 1 [file NIHPP2026.06.09.731100v1-supplement-1.pdf]

bioRxiv preprint doi: https://doi.org/10.64898/2026.06.09.731100; this version posted June 11, 2026. The copyright holder for this preprint (which was not certified by peer review) is the author/funder, who has granted bioRxiv a license to display the preprint in perpetuity. It is made available under a CC-BY 4.0 International license.

Supplementary Information for

Mutant SOD1 expressed by oligodendrocytes aggregates in myelinic nanochannels and accelerates disease progression in familial ALS mice

Alexandra I. Mot, Ying Li, Payam Dibaj, Iva D. Tzvetanova, Ulrike C. Gerwig, Tizibt A. Bogale, Sandra Goebbels, Wiebke Möbius, Dwight E. Bergles, Brett M. Morrison, Jeffrey D. Rothstein, Don W. Cleveland, Julia M. Edgar and Klaus-Armin Nave.

Corresponding authors:
Don W. Cleveland (dcleveland@health.ucsd.edu)
Klaus-Armin Nave (nave@mpinat.mpg.de)

This PDF file includes:

Supplementary text

References for SI reference citations

bioRxiv preprint doi: https://doi.org/10.64898/2026.06.09.731100; this version posted June 11, 2026. The copyright holder for this preprint (which was not certified by peer review) is the author/funder, who has granted bioRxiv a license to display the preprint in perpetuity. It is made available under a CC-BY 4.0 International license.

Supplementary Information

SI Material and Methods

Mouse models and husbandry

Animals used for experiments shown in Figures 1, 2, 3, and 5 were housed at the Max Planck Institute for Multidisciplinary Sciences (City Campus) animal facility and all experiments were conducted in compliance with protocols approved by the local German authorities (Niedersächsisches Landesamt für Verbraucherschutz und Lebensmittelsicherheit). Animals used for experiments shown in Figure 4 were housed at the Johns Hopkins University animal facility and all experiments were conducted in compliance with protocols approved by the Animal Care and Use Committee at the Johns Hopkins University. SOD1-G93A (*1*), loxSOD1^{G37R} (*2*), Sox10-Cre (*3*), MOGi-Cre (*4*), CNPnull (*5*), and PLP-eGFP (*6*) mice were generated and described previously. All animals were maintained as heterozygotes or hemizygotes, except for CNPnull mice that were maintained as homozygotes. SOD1-G93A, loxSOD1^{G37R} and PLP-eGFP mice were maintained in a mixed background. Sox10-Cre, CNPnull and MOGi-Cre mice were in a C57BL/6 background before being bred with SOD1-G93A or loxSOD1^{G37R} mice. No gender differences in disease development were observed in our studies nor reported by other investigators (*2, 7, 8*). Animals were excluded from the study if they developed severe fight wounds or dermatitis, requiring their immediate euthanasia. All mice were group-housed in individually ventilated cages and kept under a 12:12 light/dark cycle (Max Planck Institute) or 14:10 light/dark cycle (Johns Hopkins University) with access to food ad libitum. For genotyping, genomic DNA was isolated from tail or ear clip biopsies and subjected to routine PCR methods.

Mouse behavioral testing and phenotyping

For experiments involving SOD1-G93A and loxSOD1^{G37R} mice, the time of disease onset, early disease and endstage were defined as the time when mouse body weight reached the peak, body weight declined to 10% of the maximum weight and paralyzed mice could not right themselves within 20 seconds when placed on their side, respectively, as described previously (*2*). LoxSOD1^{G37R} mice were assessed for neurological score on a scale from 0–2 as follows: 0, no impairment; 0.5, slight hindlimb weakness; 1, tremor/ataxia; 1.5, hindlimb muscle tone changes; 2, endstage. SOD1-G93A mice were assessed for neurological score on a scale from 0–2 as follows:

bioRxiv preprint doi: https://doi.org/10.64898/2026.06.09.731100; this version posted June 11, 2026. The copyright holder for this preprint (which was not certified by peer review) is the author/funder, who has granted bioRxiv a license to display the preprint in perpetuity. It is made available under a CC-BY 4.0 International license.

0, no impairment; 0.5, tremor/ataxia; 1, hindlimb muscle tone changes; 1.5, moderate paralysis; 2, endstage. For experiments involving loxSOD1^{G37R} mice, motor performance was evaluated by means of the rotarod test. Mice were placed on a horizontal rod that rotated at an accelerating speed (1 rpm every 10 seconds, up to a maximum of 26 rpm) and the time it took for the mouse to fall off was recorded. Mice were tested at 7-day intervals and each mouse was tested three times per trial and the average time was used.

Primary oligodendrocyte cultures and immunocytochemistry

Primary oligodendrocyte cultures were prepared as previously described (*9, 10*). Briefly, postnatal day 0-2 (P0-2) cortices from hemizygous mutant SOD1-G93A mice were dissected, dissociated using Papain, and grown as mixed glial cultures in DMEM (Lonza) supplemented with 10% fetal calf serum (FCS), GlutaMAX (Invitrogen), and Penicillin and Streptomycin (Invitrogen) for 12-16 days. OPCs were isolated using mechanical dissociation followed by 20 min differential adhesion on uncoated petri dishes. Remaining microglia were removed by three rounds of immunopanning on dishes coated with 2.3µg/ml BS-lectin-1 in Dulbecco's phosphate buffered saline (PBS). Purified OPCs were differentiated for 12 days in Sato's Medium. Differentiated oligodendrocytes were fixed with 4% paraformaldehyde (PFA) for 10 min, permeabilized in 0.1% Triton X-100, washed with PBS, blocked in a solution containing 10% donkey serum and 0.1% Triton X-100 in PBS, and incubated with primary antibodies directed against CNPase (Sigma-Aldrich Cat# C9743, RRID:AB_1840761) and misfolded human SOD1 (MediMabs Cat# MM-0070-P, RRID:AB_2909641) overnight at +4°C. All primary and secondary antibodies were incubated in a blocking solution containing 10% donkey serum and 0.1% Triton X-100 in PBS. Cells were washed three times in PBS before and after secondary antibody incubation. Cells were coverslipped using Fluoromount G (Invitrogen) mounting media and imaged using confocal microscopy.

Immunoelectron microscopy

Immunogold labeling of cryosections was prepared as previously described (*11, 12*). Briefly, spinal cords from 5 month old WT and SOD1-G93A mutant mice were dissected and immersion fixed in 4% formaldehyde and 0.2% glutaraldehyde in 0.1 M phosphate buffer containing 0.5% NaCl. After vibratome sectioning (VT1200S, Leica Microsystems, Wetzlar, Germany) pieces of cortical spinal tract and ventral regions with cell bodies of motor neurons were cryo-protected by infiltration with 2.3M sucrose in 0.1M phosphate buffer, placed on aluminum pins and then frozen in liquid

bioRxiv preprint doi: https://doi.org/10.64898/2026.06.09.731100; this version posted June 11, 2026. The copyright holder for this preprint (which was not certified by peer review) is the author/funder, who has granted bioRxiv a license to display the preprint in perpetuity. It is made available under a CC-BY 4.0 International license.

nitrogen. Ultrathin cryosections sections were cut using an UC6 ultramicrotome (Leica Microsystems, Wetzlar, Germany) and a 35° cryo-immuno-diamond knife (Diatome, Biel, Switzerland). Primary antibody directed against SOD1 (Enzo Life Sciences Cat# ADI-SOD-100-D, RRID:AB_2039583) was used. Protein A-gold conjugates were used to allow for the detection of SOD1-gold labelling (Cell Microscopy Center, Department of Cell Biology, University Medical Center Utrecht, The Netherlands). Sections were imaged using a transmission electron microscope LEO912 (Carl Zeiss Microscopy, Oberkochen, Germany) and digital micrographs were taken using a 2048x2048 CCD camera (TRS, Moorenweis, Germany).

Immunohistochemistry

For experiments involving loxSOD1^{G37R} mice, 44 week old mice were anesthetized with a lethal dose of avertin and transcardially perfused with ice-cold Hank's buffered salt solution followed by 4% PFA in 0.1 M phosphate buffer, pH 7.4. Spinal cords were dissected and post-fixed overnight in 4% PFA and phosphate buffer. Fixated spinal cords were subjected to dehydration steps (50% ethanol, 80% ethanol, 100% ethanol, 100% isopropanol, 50% isopropanol and 50% xylol, twice 100% xylol) followed by paraffinization on a STP 120 Spin Tissue Processor (Leica Microsystems). Samples were embedded in paraffin blocks on a HistoStar embedding workstation (Epredia). Paraffin-embedded blocks of lumber spinal cord were sectioned transversely in 5-µm slice thickness. Slices were mounted onto slides and dried overnight. Slides were deparaffinized at 60 °C followed by incubation in xylol (100% twice) and a 1:1 mixture of xylol and isopropanol (once) for 10 min each. The slides were rehydrated in a descending ethanol series (100% ethanol, 90% ethanol, 70% ethanol, 50% ethanol, and water) for 5 min each. This was followed by incubation in sodium citrate buffer (0.01 M, pH 6.0) for 5 min at RT and boiling for 10 min. The samples were cooled for 20 min and washed in Tris buffer with 2% milk powder for 5 min. Subsequently, slides were blocked for 20 min at RT in 20% goat serum (BSA/PBS) and incubated at +4°C overnight with primary antibodies directed against MBP (rabbit, custom-made in the Nave Laboratory, 1:5,000) and misfolded human SOD1 (mouse, MediMabs Cat# MM-0070-P, RRID:AB_2909641, 1:500). Slides were then washed 3 times for 5 min each in Tris buffer with 2% milk powder. Slides were incubated for 1 hour at RT in the dark with DAPI (Thermo-Fisher, 1:20000) and the corresponding fluorescent secondary antibodies: anti-mouse Alexa555 (donkey/goat, Thermo-Fisher; 1:1,000) and anti-rabbit Dylight633 (donkey/goat, Thermo-Fisher; 1:1,000). Slides were again washed 3 times for 5 min each in the dark using Tris buffer. Then slides

bioRxiv preprint doi: https://doi.org/10.64898/2026.06.09.731100; this version posted June 11, 2026. The copyright holder for this preprint (which was not certified by peer review) is the author/funder, who has granted bioRxiv a license to display the preprint in perpetuity. It is made available under a CC-BY 4.0 International license.

were mounted with Aqua PolyMount mounting medium (PolySciences) and allowed to dry overnight at +4°C in the dark. Mounted slides were imaged using an epifluorescence microscope (Zeiss Axio Imager Z1) and Zen software (Zeiss) using appropriate excitation and emission filters. Quantification of misfolded SOD1 +ve staining was calculated by performing thresholding and the percentage of positive area was determined using Fiji in the respective ROIs (grey matter and white matter).

Myelin purification

Purification of the myelin enriched fraction was performed as previously described (*13*). For experiments involving loxSOD1^{G37R} mice, 44 week old mice were killed by cervical dislocation, full spinal cords were dissected out and fresh-frozen on dry ice. Tissues were homogenized in 0.32 M sucrose with protease inhibitor and carefully layered on top of a 0.85 M sucrose solution and then centrifuged for 30 min at 75,000 × g at +4°C. The interphase, containing crudely purified myelin, was carefully collected, resuspended in water, and centrifuged for 15 min at 75,000 × g at 4 °C. The pellet was subjected to osmotic shock by resuspension in water for at least 10 min followed by centrifugation for 15 min at 12,000 × g at +4°C. After a second osmotic shock performed as above, the pellet was resuspended in 0.32 M sucrose solution containing protease inhibitor, carefully added on top of a 0.85 M sucrose solution, and centrifuged for 30 min at 75,000 × g at +4°C. The interphase containing purified myelin was carefully collected, resuspended in water, and centrifuged for 15 min at 75,000 × g. The pellet was resuspended in 1×TBS (supplemented with protease inhibitor, Complete Mini, Roche), and stored at −80°C.

Western blotting

To determine the protein concentration in purified myelin samples, the DC protein assay (BioRad Laboratories) was used according to the manufacturer's guidelines. Samples were then mixed with sample buffer to a final concentration of 2% (w/v) LDS, 0.125% (v/v) Triton X-100, 0.125% (w/v) sodium deoxycholate, 0.501 mM EDTA, 10% glycerol, 247 mM Tris–HCl (pH 8,4), 0.01875% (w/v) Coomassie G250, 0.00625% (w/v) phenol red, 25 mM DTT and 1 µg/µL total protein and heated at +40°C (with shaking at 750 rpm) for 10 min. 15 µg total protein was loaded per lane and separated on 12% Tris-Glycine SDS-PAGE gels in Tris-Glycine SDS running buffer for 1.5 h at 120 V using the Mini protean chamber (Biorad). Proteins were transferred onto a PVDF Membrane (Immobilon-FL PVDF, IPFL00010, Merck Millipore) in transfer buffer (25 mM Tris, 192 mM

bioRxiv preprint doi: https://doi.org/10.64898/2026.06.09.731100; this version posted June 11, 2026. The copyright holder for this preprint (which was not certified by peer review) is the author/funder, who has granted bioRxiv a license to display the preprint in perpetuity. It is made available under a CC-BY 4.0 International license.

glycine, 10% methanol) using a XCell II™ Blot Module (Thermofisher) for 1 h at 38 V. To verify equal loading, blots were stained by fast green (5 mg/L fast green, Sigma, in 6.7% acetic acid and 30% methanol) immediately after transfer for 5 min in the dark, washed twice for 30 sec in 6.7% acetic acid and 30% methanol, and imaged using a ChemoStar fluorescent imager (Intas) equipped with a 670 nm/20 nm excitation filter and near-infrared emission collection. Blots were destained in 50% ethanol in TBS-T (150 mM NaCl, 10 mM Tris/HCl [pH 7.4], 0.05% Tween-20) twice for 5 min each and then washed twice in distilled water for 30 sec each. Membranes were blocked in 5% skim milk in TBS-T for 1 h at RT on a rotating shaker. Membranes were then incubated in primary antibody directed against human and mouse SOD1 (Enzo Life Sciences Cat# ADI-SOD-100-D, RRID:AB_2039583, 1:1000) overnight at +4°C on a rotating shaker. After washing with TBS-T, blots were incubated with the secondary antibody anti-rabbit IgG (H+L)-HRPO MinX none (Dianova, Cat# 111-035-003, 1:5,000) for 2 h at RT on a rotating shaker. After washing with TBS-T, blots were detected using WesternBright Sirius HRP substrate (Advansta, Cat# K-12043) and the Odyssey platform (Licor). For quantification of protein abundance, bands were analyzed using Fiji. Protein levels were normalized to fast green total protein.

RNA extraction and quantitative PCR

To determine Cre recombination efficiency *in vivo*, spinal cord neural cells from 1.5-month-old loxSOD1^{G37R}; PLP-eGFP and MOGi-Cre; loxSOD1^{G37R}; PLP-eGFP mice were dissociated using Miltenyi Dissociation Kit (P) following the manufacturer's instructions. Myelin was removed by using myelin removal beads (Miltenyi). eGFP+ cells were sorted by fluorescence-activated cell sorting (FACS) using MoFlo MLS high-speed cell sorter (Beckman Coulter) at Johns Hopkins School of Public Health FACS core. Dead cells were excluded by propidium iodide staining during sorting. Genomic DNA was extracted from cultured or FACSed cells using QIAmp DNA micro kit (Qiagen) and quantitative PCR for *SOD1* transgene was performed using the primers and cycler parameters on ABI Plusone cycler, as described previously (*2*).

Statistical analysis

The group size (number of animals = n) and the statistical tests used are indicated in the respective figure legend. Graphs represent means, and errors are reported as the standard error of the mean (SEM). To account for missing values due to disease progression in neurological score and rotarod data, area under the curve was calculated for each animal and groups were compared using one-

bioRxiv preprint doi: https://doi.org/10.64898/2026.06.09.731100; this version posted June 11, 2026. The copyright holder for this preprint (which was not certified by peer review) is the author/funder, who has granted bioRxiv a license to display the preprint in perpetuity. It is made available under a CC-BY 4.0 International license.

way ANOVA with Bonferroni post hoc. All statistical analyses were performed using GraphPad Prism 7. $P < 0.05$ was considered to be statistically significant.

References

1. M. E. Gurney *et al.*, Motor neuron degeneration in mice that express a human Cu,Zn superoxide dismutase mutation. *Science* **264**, 1772-1775 (1994).
2. S. Boillée *et al.*, Onset and progression in inherited ALS determined by motor neurons and microglia. *Science* **312**, 1389-1392 (2006).
3. T. Matsuoka *et al.*, Neural crest origins of the neck and shoulder. *Nature* **436**, 347-355 (2005).
4. N. Hövelmeyer *et al.*, Apoptosis of oligodendrocytes via Fas and TNF-R1 is a key event in the induction of experimental autoimmune encephalomyelitis. *J Immunol* **175**, 5875-5884 (2005).
5. C. Lappe-Siefke *et al.*, Disruption of Cnp1 uncouples oligodendroglial functions in axonal support and myelination. *Nat Genet* **33**, 366-374 (2003).
6. B. S. Mallon, H. E. Shick, G. J. Kidd, W. B. Macklin, Proteolipid promoter activity distinguishes two populations of NG2-positive cells throughout neonatal cortical development. *J Neurosci* **22**, 876-885 (2002).
7. K. Yamanaka *et al.*, Astrocytes as determinants of disease progression in inherited amyotrophic lateral sclerosis. *Nat Neurosci* **11**, 251-253 (2008).
8. S. H. Kang *et al.*, Degeneration and impaired regeneration of gray matter oligodendrocytes in amyotrophic lateral sclerosis. *Nat Neurosci* **16**, 571-579 (2013).
9. C. S. Barros *et al.*, Beta1 integrins are required for normal CNS myelination and promote AKT-dependent myelin outgrowth. *Development* **136**, 2717-2724 (2009).
10. A. S. Saab *et al.*, Oligodendroglial NMDA Receptors Regulate Glucose Import and Axonal Energy Metabolism. *Neuron* **91**, 119-132 (2016).
11. M. T. Weil, T. Ruhwedel, M. Meschkat, B. Sadowski, W. Möbius, Transmission Electron Microscopy of Oligodendrocytes and Myelin. *Methods Mol Biol* **1936**, 343-375 (2019).
12. P. J. Peters, J. Pierson, Immunogold labeling of thawed cryosections. *Methods Cell Biol* **88**, 131-149 (2008).

bioRxiv preprint doi: https://doi.org/10.64898/2026.06.09.731100; this version posted June 11, 2026. The copyright holder for this preprint (which was not certified by peer review) is the author/funder, who has granted bioRxiv a license to display the preprint in perpetuity. It is made available under a CC-BY 4.0 International license.

13. M. S. Erwig *et al.*, Myelin: Methods for Purification and Proteome Analysis. *Methods Mol Biol* **1936**, 37-63 (2019).
